# Enhancement of Interface between Lignocellulosic Fibers and Polypropylene Matrix via the Structure Alteration of Lignin at Elevated Temperatures

**DOI:** 10.3390/ma13235428

**Published:** 2020-11-28

**Authors:** Zhen Dong, Na Li, Aixue Dong, Bomou Ma, Chongwen Yu, Teye Chu, Qixia Liu

**Affiliations:** 1 College of Textiles & Apparel, Nantong University, Nantong 226019, China; d.zhen@ntu.edu.cn (Z.D.); 2012320001@stmail.ntu.edu.cn (N.L.); 2 Key Laboratory of Cleaning Dyeing and Finishing Technology of Zhejiang Province, Shaoxing University, Shaoxing 312000, China; aixue_dong@126.com; 3 College of Textiles, Donghua University, Shanghai 201620, China; mabomou@dhu.edu.cn (B.M.); yucw@dhu.edu.cn (C.Y.); 4 Research and Development Centre, Jiangxi Enda Technology Co., Ltd., Xinyu 336600, China; chuteye@163.com

**Keywords:** lignocellulose fiber, cellulose, interface, lignin, natural fiber

## Abstract

This paper investigated the feasibility of enhancing the interface between lignocellulosic fibers and a polypropylene matrix via structure alteration of lignin at elevated temperatures. Alkali treatment can remove gum substances from lignocellulose fibers effectively at elevated temperatures but easily causes damages to fiber strength. In previous studies on directional delignification of lignocellulosic fibers, loss of fiber strength is avoided but condensation and degradation of lignin are accelerated. So far, few reports have been available on the effect of lignin structures on the interface between fibers and a matrix. In this study, jute fibers with different lignin structures are produced at 100 and 130 °C for reinforcing a polypropylene matrix. The interface between the fibers and matrix is analyzed. The result shows that decrease in aliphatic hydroxyl concentration by 9.5% at 130 °C from 3 to 5 h contributes to a 14.2% decrease in the surface energy of jute fibers. Meanwhile, the polydispersity index of lignin decreases from 1.21 to 1.15. Centralized distribution of lignin molecule-weight and reduction in fiber surface energy improves the interface between the fibers and matrix, which manifests as a 30.8% increase in the impact strength of the composites. Similar improvement is not observed in the composites reinforced with jute fibers at 100 °C, due to the absence of lignin-structure changes. This paper provides a new strategy to improve the interface between lignocellulose fibers and a hydrophobic matrix.

## 1. Introduction

Lignocellulosic fibers are widely used to reinforce polymer matrices but are limited in high performance applications due to their hydrophilic nature [1]. Natural lignocellulosic fibers are cheap, renewable and available in abundance, which makes them a competitor to glass or carbon fibers in semi-structural use products, e.g., furniture, automotive and building products [2,3,4]. However, most natural lignocellulosic fibers have moisture regain as high as 12.6% due to existence of hydrophilic gum substances. It is reported that jute fibers have 12–13 wt % of lignin, 13.6–20.4 wt % of hemicellulose and 0.2 wt % of pectin [3]. These hydrophilic substances may destroy the interface between the fibers and hydrophobic matrix, resulting in composites with limited mechanical properties.

Alkaline pretreatment methods are commonly used in modification of lignocellulose fibers for composites [5]. In the latest research, silane coupling agents [6], hybrid compatibilizers [7], rare earth compounds [8] and ionic liquids [9] are found to be effective in improving the interface between the lignocellulosic fibers and matrix. However, taking into account factors including cost and ease of use, alkali treatment is most widely used. Partial glycoside bonds and ester-type linkages between lignin and hemicellulose units are sensitive to alkali [3,10]. After alkaline pretreatment, a certain amount of lignin, polysaccharide and wax are removed, which may easily cause damage to fiber strength [3,11,12]. Therefore, most alkaline pretreatments choose mild temperature conditions [13,14,15]. In our previous studies, a directional delignification method is studied to overcome the shortcomings of traditional alkaline pretreatments [16]. Glycerol is chosen as a solvent of alkali and as a heating medium in modification of lignocellulosic fibers. With the assistance of microwaves, glycerol and alkali on the fiber surface can be heated quickly to temperatures above 100 °C. Meanwhile, permeation of alkali molecules in the radial direction of lignocellulosic fibers is inhibited due to high solvent viscosity. In this case, delignification behavior is limited in the outer layer and loss of fiber strength is avoided.

Lignin plays an important role in the interface between the lignocellulosic fibers and polymer matrix. In general, lignin is difficult to be removed thoroughly from fibers even after severe alkaline pretreatments [17]. Therefore, changes of lignin in fibers will definitely affect adhesion between the fibers and matrix. Nowadays, most studies on pretreatment of lignocellulose fibers focus on removal rather than alteration of lignin structures [18,19,20]. Zhao et al. found that depolymerization and condensation reactions of lignin were accelerated simultaneously in a hydrothermal environment, which might improve self-bonding of natural fiber materials [21]. Therefore, it seems feasible to enhance the interface of lignocellulose-fiber reinforced composites via alteration of lignin structures at elevated temperatures.

This paper aims to study structure changes of lignin at elevated temperatures and their effect on the interface between the lignocellulosic fibers and matrix. In the directional delignification method, it’s difficult to determine the change of lignin structure quantitatively, since delignification behavior is limited only in the outer layer. In this study, raw jute fibers are pretreated in alkaline aqueous solutions under high temperatures for structural analysis of lignin. The effect of lignin-structure changes on the interface between the fibers and matrix is analyzed.

## 2. Materials and Methods

### 2.1. Materials

Raw jute fibers with a fineness of 40 dtex and an average length of 124.5 mm are provided by Enda Group, Xinyu, China. Polypropylene fibers with a melting point of 165 °C and a density of 0.9 g/cm^3^ are purchased from New Green Leaves Nonwoven Co., Ltd, Nantong, China. Sodium hydroxide and other reagents are analytical grade and purchased from Runjie Co., Ltd., Shanghai, China.

### 2.2. Alkaline Pretreatment of Jute Fibers

Raw jute fibers were pretreated by alkali at elevated temperatures from 1 to 5 h with a liquor to fiber ratio of 30:1 (V:W). A commonly used alkali concentration of 8 wt % is selected in this study to improve the hydrophobicity and fineness of jute fibers [18]. Considering that lignocellulosic fibers are easily disintegrated into single cells at temperatures above 130 °C [17], two representative temperatures, 100 and 130 °C, are chosen in the alkaline pretreatments. Afterwards, all fibers were rinsed thoroughly with water and dried at room temperature.

### 2.3. Fabrication of Fiber Reinforced Composites

The composites were prepared using a compression molding method reported by Hou et al. [22]. Firstly, jute fibers after alkaline pretreatment were blended with polypropylene fibers using a mass ratio of 30:70 and then processed into a fibrous mat on an FN300A model roller carding machine (Qingdao Jingjia Co. Ltd., Shandong, China). Afterwards, the mat was compressed at 120 °C under 12 MPa for 6 min to remove the air between the fibers. Finally, the fibrous mat was compression molded into composites at 195 °C under 18 MPa for 20 min. In this study, composites of raw jute fibers were used as the control.

### 2.4. Determination of Lignin Content

The content of Klason lignin was determined according to ASTM standard D1106-96. All data were tested three times and given as mean ± standard deviation.

### 2.5. Isolation and Purification of Milled Wood Lignin

Isolation and purification of milled wood lignin (MWL) were carried out according to the method reported by Björkman [23] and Lundquist et al. [24], respectively.

### 2.6. Characterization of Lignin Structures

#### 2.6.1. Molecular Weight and Molecular Weight Distribution

The weight-average molecular weight (M_w_) and number-average molecular weight (M_n_) were determined using a Waters 1515 Isocratic HPLC (Waters Inc., Milford, CT, USA). About 10 mg of lignin was dissolved in 2 mL of tetrahydrofuran, then about 20 µL of lignin solution was injected into the HPLC column. The molecular weight and molecular weight distribution were determined using standard polystyrene as a benchmark. All molecule-weight data were tested 3 times and given as mean ± standard deviation.

#### 2.6.2. Ratio of Condensed Units

The ratio of condensed lignin was determined using 2D nuclear magnetic resonance (NMR) of MWL. About 270 mg of MWL was dissolved in 0.4 mL of dimethyl sulfoxide-d6, and then 2D NMR was recorded at 400 MHz on a Bruker AV III NMR (Bruker, Switzerland) spectrometer using HSQC mode. The condensation degree of MWL was defined as the ratio of condensed units, i.e., the number of lignin units with β-5 and 5-5 linkages divided by the number of phenylpropane units. According to the method of Sette et al. [25], phenylpropane units were quantified by total integration of half syringyl signal (S_2,6_) plus G_2_. All data were determined three times and given in the form of mean and standard deviation.

#### 2.6.3. Hydroxyl Concentration

The number of hydroxyl groups per C_9_ unit is determined based on integration of 1H NMR spectrum. About 10 mg of acetylated lignin was dissolved in 0.4 mL of CDCl_3_. The 1H NMR was recorded at 400 MHz on a Bruker AV III NMR spectrometer. The integration region from 2.5 to 2.22 pm, and 2.22 to 1.6 pm corresponds to aromatic hydroxyl and aliphatic hydroxyl, respectively. The C_9_ formula of lignin molecule was determined according to the method described by Yan et al. [26].

### 2.7. Tensile Strength at Break of Jute Fibers

The tensile strength at break of jute fibers was determined according to ASTM D 3822-07 on a 3385H model material testing machine (Instron Inc., Norwood, MA, USA). About 250 fibers were tested for each with a gauge length of 10 mm and a crosshead speed of 10 mm/min. All data were given in the form of mean ± standard deviation.

### 2.8. Surface Energy of Jute Fibers

The surface energy of jute fibers was measured according to the method of Owens & Wendt [27]. The contact angles of water and glycerol on the fiber surface were determined and then substituted into Equation (1) as known parameters.

(1) $$\left(1+\cos\theta \right)\gamma_{L}=2{\left(\gamma_{S}^{d}\gamma_{L}^{d}\right)}^{1/2}+2{\left(\gamma_{S}^{p}\gamma_{L}^{p}\right)}^{1/2}$$

in which θ is the contact angle of named liquids on the fiber surface. $\gamma_{L}$ represents the surface energy of the named liquids ($\gamma_{H_{2}0}$ is 72.8,${\;\gamma }_{\mathrm{Glycerol}}$ is 64.0). $\gamma_{L}^{d}$ and $\gamma_{L}^{p}$ represent the dispersion and polar component of named liquids, respectively. $\gamma_{S}^{d}$ and $\gamma_{S}^{p}$ refer to the dispersion and polar component of the tested fiber samples, respectively. All data were tested three times and given as mean ± standard deviation. Data of $\gamma_{L}^{d}$ and $\gamma_{L}^{p}$ were from the report of Jańczuk et al. [28].

### 2.9. Impact Strength of Jute Composites

The impact strength of the unnotched composite specimen was measured on an Instron universal test instrument (3385 H, Instron Co., Norwood, MA, USA) according to ISO 179-1993 standard. All data were tested three times and given in the form of mean ± standard deviation.

### 2.10. Morphological of Jute Fibers and the Fracture Sections of Jute Composites

A Gemini 300 model (ZEISS, Oberkochen, Germany) scanning electron microscope (SEM) was used to observe the morphology of jute fibers and fracture sections of jute composites. The samples were mounted on conductive adhesive tape, sputter coated with gold and observed under voltage of 5 kV.

## 3. Results and Discussion

### 3.1. Content of Lignin in Jute Fibers

**Figure 1:**
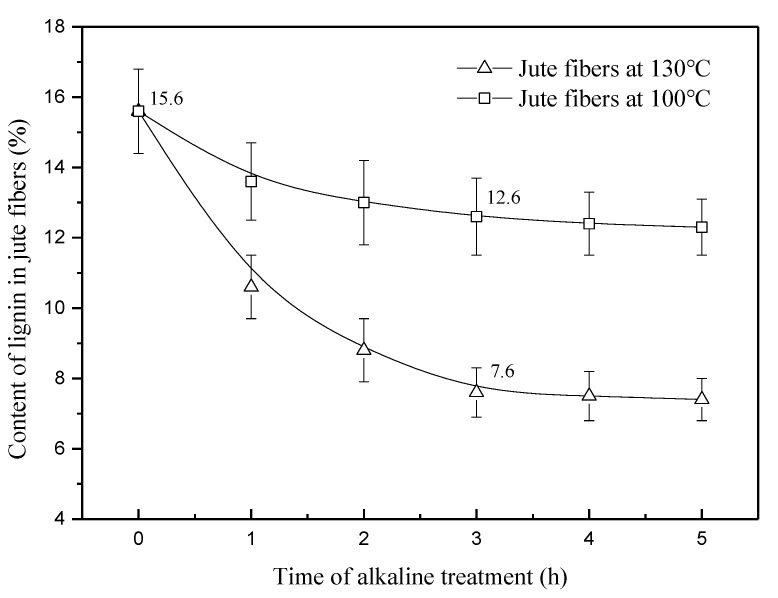
Content of lignin in the jute fibers pretreated at 100 and 130 °C.

Figure 1 shows the content of lignin in jute fibers pretreated at 100 and 130 °C. As seen in Figure 1, lignin content in both the two fibers shows a gradual decrease with time increasing from 0 to 3 h. When treatment time exceeds 3 h, the lignin content of jute fibers at 130 °C remains constant at 7.6%, far lower than 12.6% of the fibers pretreated at 100 °C. Partial glycoside bonds and ester-type linkages between the lignin and hemicellulose units are sensitive to alkali [3]. Increasing temperature may accelerate degradation and dissolution of lignin. In this study, both two fibers have lignin content that remains constant from 3 h to 5 h, which provides us a chance to study the effect of lignin structures on the interface between the fibers and matrix.

### 3.2. Structure of the Residual Lignin in Jute Fibers

#### 3.2.1. Molecular Weight

**Figure 2:**
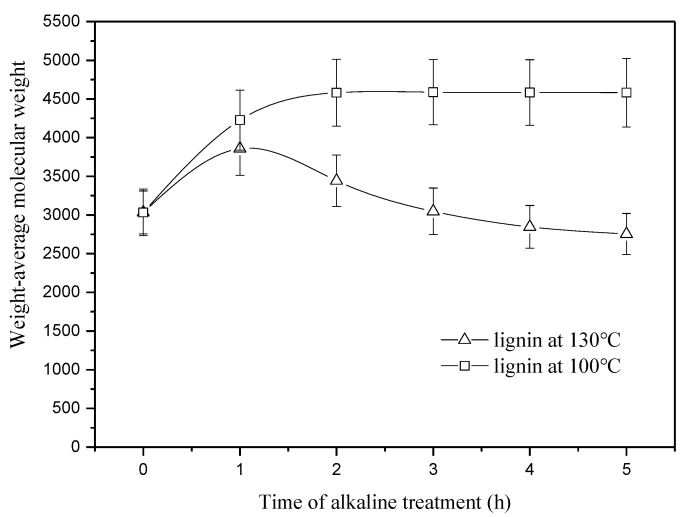
Weight-average molecular weight of residual lignin in the jute fibers pretreated at 100 and 130 °C.

Figure 2 shows the weight-average molecular weight of the residual lignin in the fibers pretreated at 100 and 130 °C. As seen in Figure 2, the molecular weight of lignin at 100 °C increases by 50% as time increases from 0 to 2 h, then remains constant at about 4589. The initial increase at from 0 to 2 h should be attributed to removal of lignin with low molecular weight. In general, lignin with low molecular weight is relatively easy to be dissolved whereas high molecule-weight fractions are limited. Subsequent constancy of molecular weight from 2 to 5 h indicates that no significant lignin degradation occurs at 100 °C. At 130 °C, the molecular weight of lignin shows a gradual decrease after an initial increase by 27.2% from 0 to 1 h. At any moment from 1 to 5 h, the molecular weight of lignin at 130 °C is always lower than that at 100 °C, suggesting that degradation of lignin at 130 °C is more pronounced than that at 100 °C. Natural lignin macromolecules have tridimensional reticulate structures, which makes them difficult to be degraded in alkaline solutions. Increasing temperature may accelerate degradation of these lignin macromolecules.

#### 3.2.2. Condensation Degree

**Figure 3:**
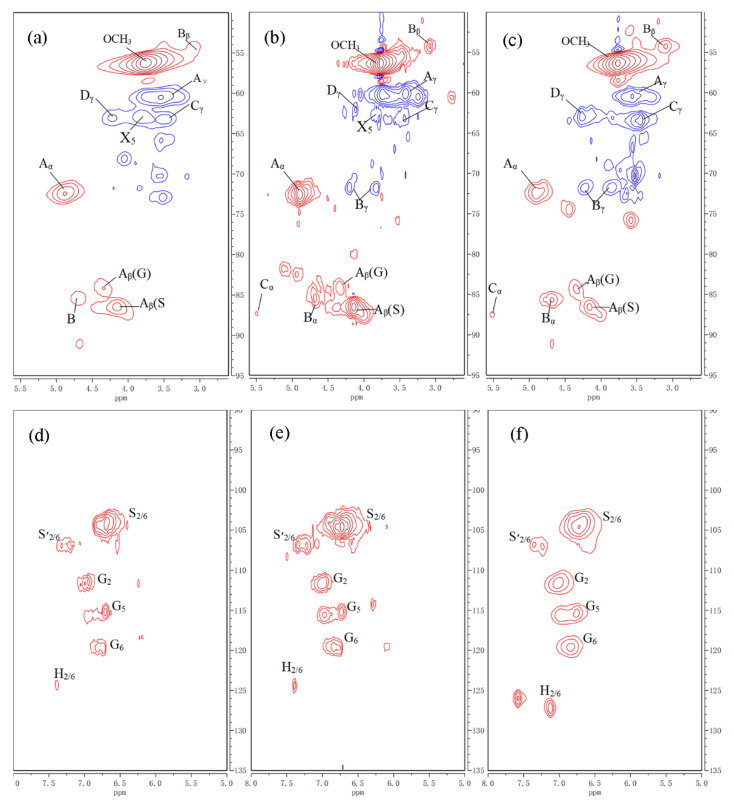
Aliphatic region in the 2D NMR spectrum of lignin from (**a**) raw jute fibers, (**b**) jute fibers pretreated at 100 °C for 5 h and (**c**) jute fibers pretreated at 130 °C for 5 h; aromatic region in the 2D NMR spectrum of lignin from (**d**) raw jute fibers, (**e**) jute fibers pretreated at 100 °C or 5 h and (**f**) jute fibers pretreated at 130 °C or 5 h.

**Figure 4:**
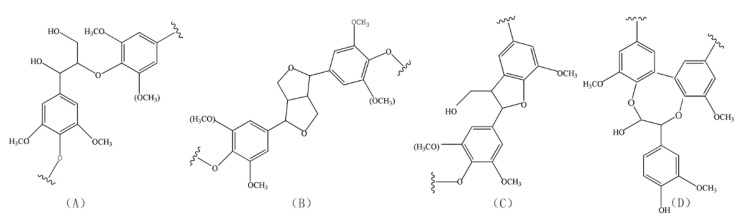
Main substructures of lignin inside the jute fibers: (**A**) β-O-4 aryl ether linkages with a free OH at the γ-carbon (**B**) resinol substructures formed by β-β, α-O-γ and γ-O-α linkages; (**C**) phenylcoumarane substructures formed by β-5 and α-O-4 linkages; (**D**) 5-5′-O-4 linkages.

Figure 3 shows the aliphatic region and aromatic region in the 2D NMR spectrum of lignin from raw jute fibers and the fibers pretreated at 100 and 130 °C. As seen in Figure 3, lignin from jute fibers mainly consists of four units, A, B, C and D, whose structures are shown in Figure 4. As seen in Figure 4, C and D structures are typical condensed units, in which β-5 and 5-5 are corresponding condensation bonding, respectively. As seen in Figure 3, the proportion of C_γ_ and D_γ_ units at 130 °C is much higher than that at 100 °C, which indicates that the condensation reaction of lignin accelerates as temperature increases from 100 to 130 °C.

**Figure 5:**
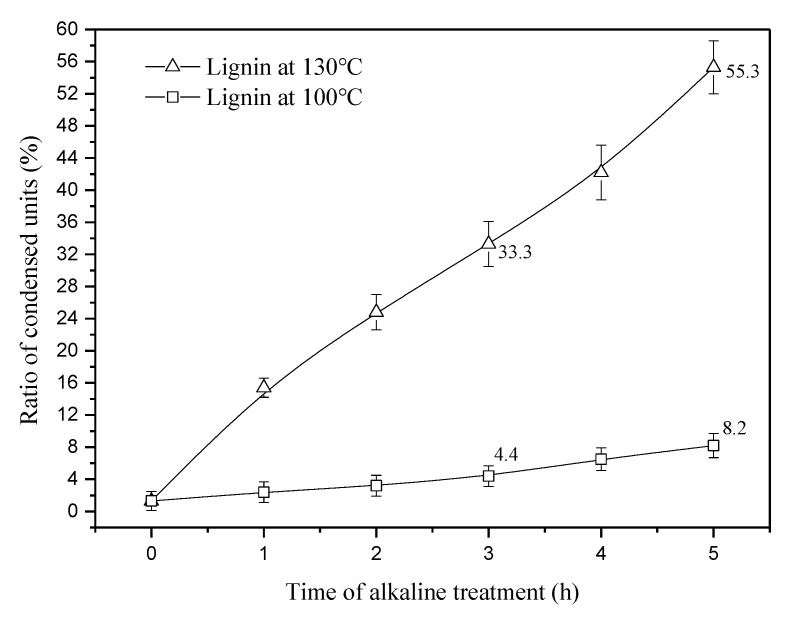
Ratio of condensed lignin in jute fibers pretreated at 100 and 130 °C.

Figure 5 shows the ratio of condensed lignin in the fibers pretreated at 100 and 130 °C. As seen in Figure 5, the ratio of condensed lignin units increases continuously at 130 °C but remains at a low level at 100 °C. From 3 to 5 h, the ratio of condensed units increases by 22%, which far exceeds the 3.8% increase at 100 °C. In this study, two relatively low temperatures, 100 and 130 °C, are selected in order to avoid disintegration of long fibers. Although the condensation reaction at 130 °C is much slower than that at temperatures above 150 °C, it provides us a chance to see the structure changes of lignin via extension of time.

#### 3.2.3. Molecule-Weight Distribution

**Figure 6:**
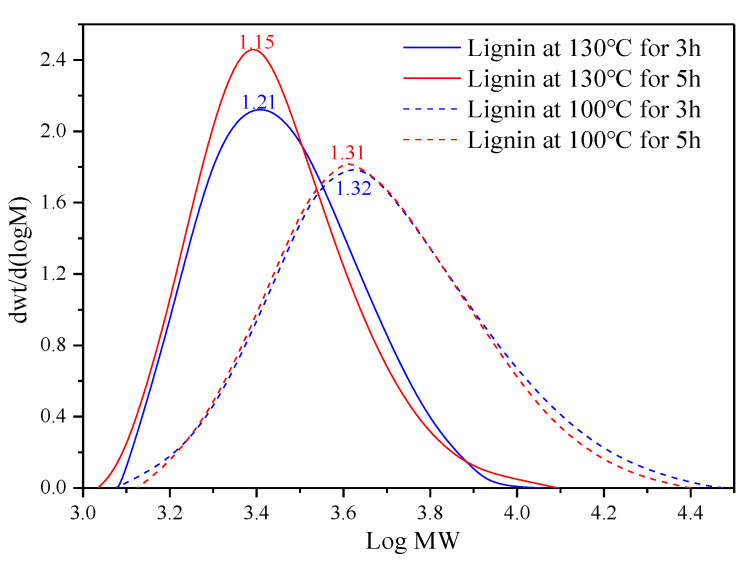
Molecule-weight distribution curves of lignin in the jute fibers pretreated for 3 and 5 h.

Degradation and condensation of lignin in fibers pretreated for 3 and 5 h cause changes in the distribution of molecular weight, as shown in Figure 6. As seen from the figure, two distribution curves of lignin from the fibers at 100 °C highly overlap, whereas lignin in the fibers at 130 °C shows different polydispersity. At a temperature of 100 °C, only lignin with low molecular weight can be dissolved. After an initial stage of massive dissolution, the structure of residual lignin remains unchanged. This phenomenon agrees well with the change of molecular weight at 100 °C shown in Figure 2. At a temperature of 130 °C, lignin macromolecules are degraded and are meanwhile accompanied with a rapid condensation reaction. The two factors work together, resulting in a reduction in the polydispersity index from 1.21 to 1.15 from 3 to 5 h. Centralized distribution of lignin molecule-weight at 130 °C may reduce weak connections in the interface between the lignocellulose fibers and polymer matrix.

#### 3.2.4. Hydroxyl Concentration

Table 1 shows the hydroxyl concentration of lignin in the fibers pretreated for 3 and 5 h. As seen in Table 1, jute fibers pretreated at 130 °C for 5 h have lignin with aliphatic hydroxyl concentration 9.5% lower, phenolic hydroxyl concentration 2.4% higher and total hydroxyl concentration 5.8% lower than the fibers pretreated at 130 °C for 3 h. β-O-4 linkages between the phenylpropane units are broken at 130 °C, resulting in an increase in the phenolic hydroxyl concentration from 3 to 5 h. Meanwhile, aliphatic hydroxyl concentration shows a decline by 9.5% due to speeding up of the β-formaldehyde elimination reaction [21]. Similar changes are not observed in the fibers pretreated at 100 °C, indicating that no significant structure changes occur in lignin at 100 °C.

### 3.3. Tensile Strength at Break of Jute Fibers

**Figure 7:**
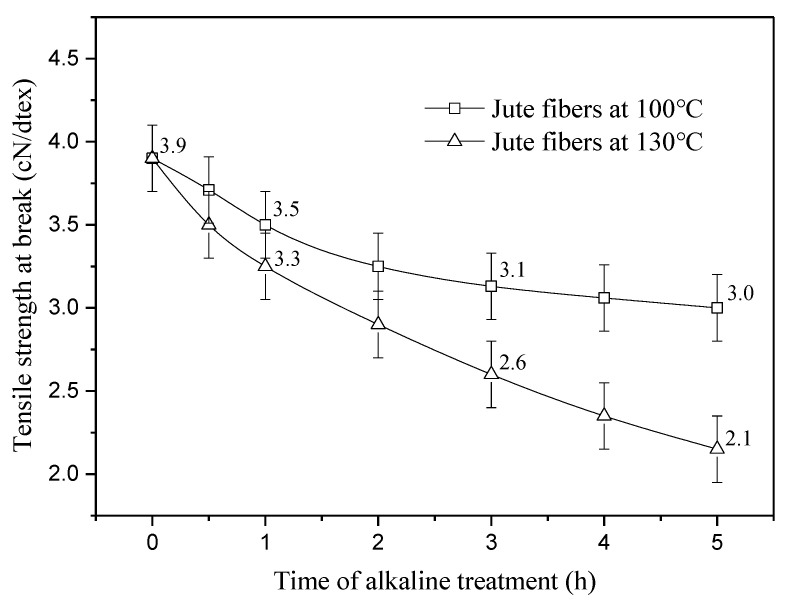
Tensile strength at break of jute fibers pretreated at 100 and 130 °C.

Figure 7 shows the tensile strength at break of jute fibers pretreated at 100 and 130 °C. As seen in Figure 7, the strength of jute fibers at 100 °C decreases from 3.5 to 3.1 cN/dtex from 1 to 3 h then tends to be constant, whereas the strength of jute fibers at 130 °C shows a continuous decrease from 3.3 to 2.1 cN/dtex as time increases from 1 to 5 h. At a temperature of 100 °C, degradation of lignin is limited. Dissolution of lignin ingredients is the main factor that affects fiber strength. Therefore, changes in fiber strength are highly consistent with those of lignin content at 100 °C (seen in Figure 1). At 130 °C, degradation of lignin plays a key role in decrease of fiber strength, especially from 3 to 5 h. Moreover, the peeling reaction of cellulose is also an important factor that affects fiber strength. In this study, although reduction in fiber strength is detrimental to the composites, it does not affect analysis on the interface between the fibers and matrix.

### 3.4. Morphology and Surface Energy of Jute Fibers

**Figure 8:**
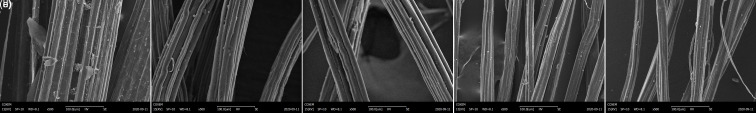
SEM images of (**a**) raw jute fibers, jute fibers pretreated at 100 °C for (**b**) 3 and (**c**) 5 h, and jute fibers pretreated at 130 °C for (**d**) 3 and (**e**) 5 h.

Figure 8 shows the morphology of raw jute fibers, jute fibers pretreated at 100 °C for 3 and 5 h, and jute fibers pretreated at 130 °C for 3 and 5 h. As seen in Figure 8a, the surface of raw jute fibers is covered with large pieces of gum substances. Part of these gum substances is removed by an alkali at 100 °C, leaving more single cells exposed on the fiber surface (seen in Figure 8b,c). At 130 °C, more gum substances are removed, resulting in fibers with a cleaner surface and better fineness (Figure 8d,e). In this study, no significant differences are observed in the surface of jute fibers pretreated for 3 and 5 h, whether at temperatures of 100 or 130 °C.

**Figure 9:**
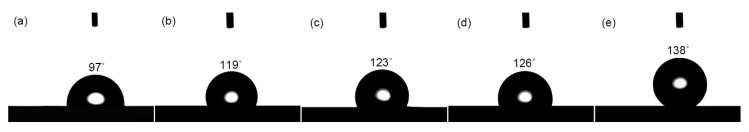
Contact angle of water on (**a**) raw jute fibers, jute fibers pretreated at 100 °C for (**b**) 3 and (**c**) 5 h, and jute fibers pretreated at 130 °C for (**d**) 3 and (**e**) 5 h.

Figure 9 shows the contact angle of water on raw jute fibers and the fibers pretreated at 100 and 130 °C. As seen in Figure 9a, raw jute fibers have a relatively small contact angle of 97°, due to existence of hydrophilic substances. Part of these hydrophilic substances is removed at 100 °C, increasing the contact angle to 119° at 3 h (Figure 9b). When the treatment time increases from 3 to 5 h, the angle increases by only 4° (seen in Figure 9c). This phenomenon suggests that alkaline pretreatment at 100 °C contributes less to the surface energy of jute fibers from 3 to 5 h. At 130 °C, the contact angle rises from 126° (Figure 9d) to 138° (Figure 9e) from 3 to 5 h. Considering that the content of lignin remains constant at this stage (seen in Figure 1), increase in the contact angle should be ascribed to the change of lignin structures. In this study, the aliphatic hydroxyl concentration at 130 °C is lowered by 9.5% as time increases from 3 to 5 h (seen in Table 1), which is responsible for the decrease of fibers hydrophilicity.

**Figure 10:**
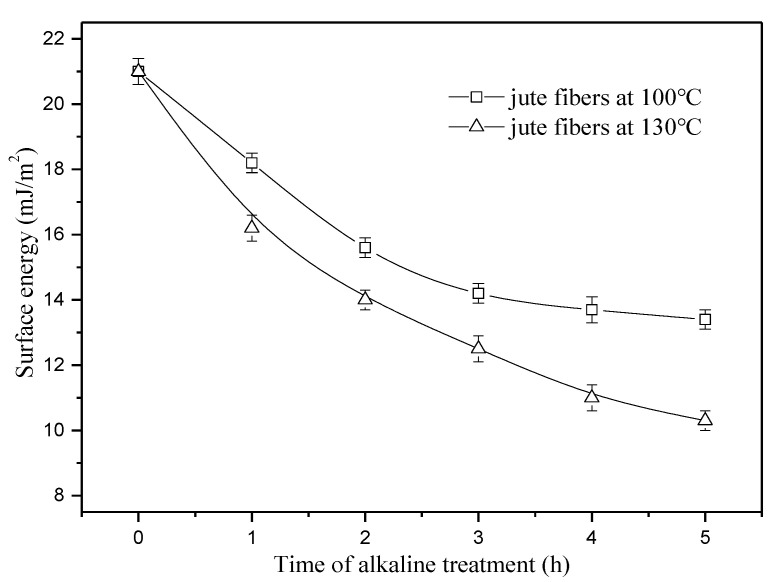
Surface energy of jute fibers pretreated at 100 and 130 °C.

Figure 10 shows the surface energy of the fibers pretreated at 100 and 130 °C. As seen in Figure 10, the surface energy of the fibers at 100 °C decreases by 32.4% as time increases from 0 to 3 h, then tends to be constant. This phenomenon is highly consistent with the change of lignin content (seen in Figure 1), suggesting that dissolution and removal of lignin is the major reason for reduction of the surface energy at 100 °C. At 130 °C, the surface energy of jute fibers decreases by 42.9% first as time increases from 0 to 3 h, and then decreases further by 14.2% from 3 to 5 h. This phenomenon agrees well with the change of water contact angle at 130 °C, shown in Figure 9. In this study, decrease in the aliphatic hydroxyl concentration at 130 °C from 3 to 5 h reduces fiber hydrophilicity, which manifests as a 14.2% decrease in the surface energy of jute fibers.

### 3.5. Impact Strength and Interface of Fiber Reinforced Composites

**Figure 11:**
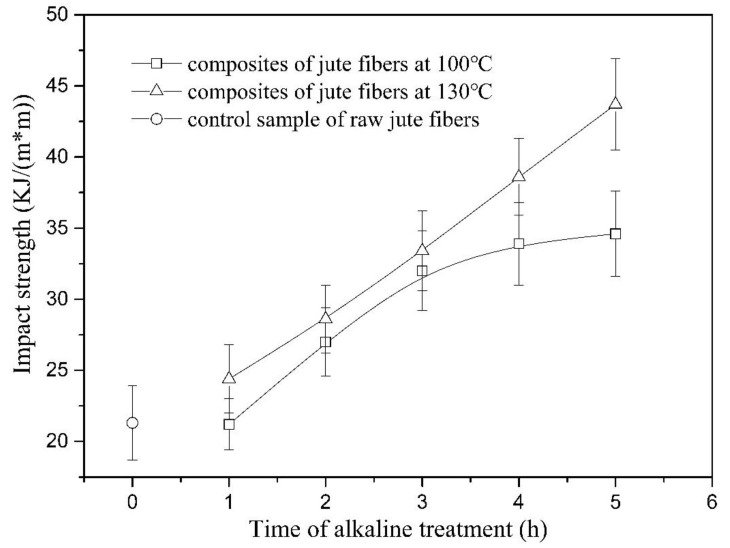
Impact strength of the composites reinforced with jute fibers pretreated at 100 and 130 °C.

Figure 11 shows the impact strength of the composites reinforced with jute fibers pretreated at 100 °C (composites_100 °C_) and 130 °C (composites_130 °C_). As seen in Figure 11, the impact strength of composites_100 °C_ increases by 50.9% as time increases from 1 to 3 h and then tends to be constant, whereas the strength of composites_130 °C_ shows a continuous increase by 79.1% from 1 to 5 h. Considering that the tensile strength at break of the fibers at 100 and 130 °C both shows a decrease from 1 to 5 h (seen in Figure 7), an increase in the impact strength of the composites should be attributed to improvement of interface between fibers and matrix [29].

**Figure 12:**
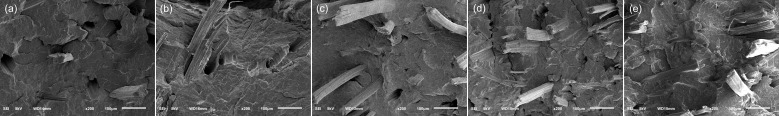
SEM images of the fracture surface of composites reinforced with (**a**) raw jute fibers, jute fibers pretreated at 100 °C for (**b**) 3 and (**c**) 5 h, and jute fibers pretreated at 130 °C for (**d**) 3 and (**e**) 5 h.

Figure 12 shows the fracture section of the composites reinforced with raw jute fibers and fibers pretreated at 100 and 130 °C. As seen in Figure 12a, raw jute fibers are easily pulled out from the matrix, due to existence of a large amount of gum substances on the fiber surface. Alkali treatment at 100 °C removed part of the hydrophilic gum substances and thus improved compatibility between the fibers and matrix, resulting in an increase in the impact strength of the composites. However, the interface adhesion between the fibers and matrix is still weak, which can be confirmed by a long pull-out length, as shown in Figure 12b. Increasing the treatment time from 3 to 5 h did not cause a significant change in pull-out length (Figure 12c), suggesting that alkaline pretreatment at 100 °C from 3 to 5 h contributes less to interface improvement. This phenomenon agrees well with the change of impact strength at 100 °C from 3 to 5 h (seen in Figure 11). At 100 °C, a certain amount of lignin is dissolved, but few changes are observed in the lignin structure. The interface between the fibers and matrix is affected only by the content of lignin. Therefore, changes in the impact strength of composite_100°C_ show a high consistence with those of the lignin content (seen in Figure 1).

As seen in Figure 12, composites of the fibers pretreated at 130 °C for 3 h (Figure 12d) have shorter pull-out length than the composite of fibers pretreated at 100 °C for 3 h (Figure 12b). This phenomenon suggests that the fibers at 130 °C have better cohesion with polypropylene than the fibers at 100 °C. At 130 °C, lignin is removed more effectively. Therefore, the fibers at 130 °C have lower surface energy and better compatibility with the polypropylene matrix than the fibers at 100 °C. As seen in Figure 12e, bonding between jute fibers and matrix becomes stronger when treatment time increases from 3 to 5 h. Considering that lignin content remains constant during this time, reinforcement of the interface should be ascribed to the change of lignin structures. In this study, aliphatic hydroxyl concentration decreases by 9.5% from 3 to 5 h, which results in a 14.2% reduction in the surface energy of jute fibers. Moreover, the polydispersity index decreases from 1.21 to 1.15. The two factors together improve the interface between the fibers and matrix, which manifests as a 30.8% increase in the impact strength from 3 to 5 h.

## 4. Conclusions

Jute fibers with different lignin structures are produced at temperatures of 100 and 130 °C, respectively, for studying the effect of lignin structures on the interface between lignocellulosic fibers and a hydrophobic matrix. At 100 °C, no significant changes are observed in lignin structures. Removal of lignin is the only factor that affects surface energy and the interface between the fibers and polypropylene matrix. At 130 °C, lignin content remains constant at 7.6% as time increases from 3 to 5 h. At this stage, lignin macromolecules are degraded, simultaneously accompanied with a rapid condensation reaction. The two factors work together, resulting in a decrease in the polydispersity index of lignin from 1.21 to 1.15. Meanwhile, aliphatic hydroxyl concentration decreases by 9.5%, leading to a 14.2% decrease in the surface energy of jute fibers. Centralized distribution of lignin molecule-weight and reduction in fiber surface energy together improve the interface between the fibers and matrix, which manifests as a 30.8% increase in the impact strength of the composites. A similar increase is not observed in composites of fibers pretreated at 100 °C from 3 to 5 h, due to the lack of lignin-structure changes. This paper provides a new strategy to improve the interface between natural lignocellulosic fibers and a hydrophobic matrix.

## Figures and Tables

**Table 1:** Hydroxyl concentrations of lignin in the jute fibers pretreated for 3 and 5 h.

Conditions	C_9_ Formula	OH_Aliphatic_/C_9_	OH_Phenolic_/C_9_	Total OH/C_9_
100℃ 3h	C_9_H_9.463_O_3.553_(OCH_3_)_0.990_	0.805	0.097	0.902
100℃ 5h	C_9_H_9.458_O_3.444_(OCH_3_)_0.992_	0.800	0.099	0.899
130℃ 3h	C_9_H_9.432_O_3.447_(OCH_3_)_0.911_	0.655	0.288	0.943
130℃ 5h	C_9_H_9.395_O_3.258_(OCH_3_)_0.903_	0.593	0.295	0.888
